# Around the Hospitals

**Published:** 1895-07-20

**Authors:** 


### AROUND THE HOSPITALS.

[NOTICE TO THE MANAGERS OF INSTITUTIONS.—Special space is now reserved for the insertion of notices of hospital meetings and festivals. The Editor requests that all notices may reach THE HOSPITAL Office, 428, Strand, at least one week before the date of each meeting.]

**St. Mary's Hospital.**—This hospital, like the majority of metropolitan hospitals, has a difficult task to secure funds to afford an unabated amount of relief to the sick poor. During the past year it is satisfactory to note that annual subscriptions have increased; it is a sign of local interest and energy on the part of the management. But on the other hand legacies, upon which the hospital unfortunately has still to rely very largely, show a marked abatement duirng the past two years, and so the institution is in difficulties. More annual subscriptions and ones of larger amount are absolutely essential. St. Mary's is one of the very few West End hospitals, situated in near proximity to a wealthy neighbourhood, and so it should not experience the difficulties which attend hospitals hidden from the eyes, of the rich in poorer parts of London. Every one of the houses in the neighbourhood should find a place in the subscription list, where there are still unfortunately many vacancies. The hospital serves a very large area, and great distress would be caused were it necessary to curtail the work. Yet unless a further £8,000 is provided annually this may take place before very long. Last year the festival dinner secured £2,838 for the hospital and £1,230 for the extension. This year the bazaar opened by H.R.H. the Princess of Wales provided much needed funds to the extent of quite £5,000. The administration, under Mr. Thomas Ryan's able guidance, are doing their part by promoting economy. The cost per occupied bed has now been brought down to £79 18s. 10d., and the cost per patient to £5 3s. 2d. During the year one bed was endowed by the Rev. Francis Jacob in memory of his sister, and 26 donations of £50 and upwards were received. The in-patients numbered 3,874, and the out-patients 19,892. The ordinary income amounted to £14,612, the ordinary expenditure to £21,878. The total income equalled £19,232, leaving a deficit of £2,828 on the ordinary expenditure. There are several convalescent and benevolent funds in connection with the hospital, all of which proved of great service during the year.

**Sydney Hospital for Sick Children.**—486 patients were under treatment; of these 33 died. In the diphtheria ward 143 were under treatment during the year, and 68 died. In the out-patient department there were 4,256 attendances.

